# Identifying Drug Effects via Pathway Alterations using an Integer Linear Programming Optimization Formulation on Phosphoproteomic Data

**DOI:** 10.1371/journal.pcbi.1000591

**Published:** 2009-12-04

**Authors:** Alexander Mitsos, Ioannis N. Melas, Paraskeuas Siminelakis, Aikaterini D. Chairakaki, Julio Saez-Rodriguez, Leonidas G. Alexopoulos

**Affiliations:** 1 Department of Mechanical Engineering, Massachusetts Institute of Technology, Cambridge, Massachusetts, United States of America; 2 Department of Mechanical Engineering, National Technical University of Athens, Athens, Greece; 3 Department of Systems Biology, Harvard Medical School, Boston, Massachusetts, United States of America; 4 Department of Biological Engineering, Massachusetts Institute of Technology, Cambridge, Massachusetts, United States of America; University of California San Diego, United States of America

## Abstract

Understanding the mechanisms of cell function and drug action is a major endeavor in the pharmaceutical industry. Drug effects are governed by the intrinsic properties of the drug (i.e., selectivity and potency) and the specific signaling transduction network of the host (i.e., normal vs. diseased cells). Here, we describe an unbiased, phosphoproteomic-based approach to identify drug effects by monitoring drug-induced topology alterations. With our proposed method, drug effects are investigated under diverse stimulations of the signaling network. Starting with a generic pathway made of logical gates, we build a cell-type specific map by constraining it to fit 13 key phopshoprotein signals under 55 experimental conditions. Fitting is performed via an Integer Linear Program (ILP) formulation and solution by standard ILP solvers; a procedure that drastically outperforms previous fitting schemes. Then, knowing the cell's topology, we monitor the same key phosphoprotein signals under the presence of drug and we re-optimize the specific map to reveal drug-induced topology alterations. To prove our case, we make a topology for the hepatocytic cell-line HepG2 and we evaluate the effects of 4 drugs: 3 selective inhibitors for the Epidermal Growth Factor Receptor (EGFR) and a non-selective drug. We confirm effects easily predictable from the drugs' main target (i.e., EGFR inhibitors blocks the EGFR pathway) but we also uncover unanticipated effects due to either drug promiscuity or the cell's specific topology. An interesting finding is that the selective EGFR inhibitor Gefitinib inhibits signaling downstream the Interleukin-1alpha (IL1α) pathway; an effect that cannot be extracted from binding affinity-based approaches. Our method represents an unbiased approach to identify drug effects on small to medium size pathways which is scalable to larger topologies with any type of signaling interventions (small molecules, RNAi, etc). The method can reveal drug effects on pathways, the cornerstone for identifying mechanisms of drug's efficacy.

## Author Summary

Cells are complex functional units. Signal transduction refers to the underlying mechanism that regulates cell function, and it is usually depicted on signaling pathways maps. Each cell type has distinct signaling transduction mechanisms, and several diseases arise from alterations on the signaling pathways. Small-molecule inhibitors have emerged as novel pharmaceutical interventions that aim to block certain pathways in an effort to reverse the abnormal phenotype of the diseased cells. Despite that compounds have been well designed to hit certain molecules (i.e., targets), little is known on how they act on an “operative” signaling network. Here, we combine novel high throughput protein-signaling measurements and sophisticated computational techniques to evaluate drug effects on cells. Our approach comprises of two steps: build pathways that simulate cell function and identify drug-induced alterations of those pathways. We employed our approach to evaluate the effects of 4 drugs on a cancer hepatocytic cell type. We were able to confirm the main target of the drugs but also uncover unknown off-target effects. By understanding the drug effects in normal and diseased cells we can provide important information for the analysis of clinical outcomes in order to improve drug efficacy and safety.

## Introduction

Target-based drug discovery is a predominant focus of the pharmaceutical industry. The primary objective is to selectively target protein(s) within diseased cells in order to ameliorate an undesired phenotype, e.g., unrestrained cell proliferation or inflammatory cytokine release. Ideally, other pathways within the diseased cells, as well as similar phenotypes in other cell types, should remain unaffected by the therapeutic approach. However, despite the plethora of new potential targets emerged from the sequencing of the human genome, rather few have proven effective in the clinic [1]. A major limitation is the inability to understand the mechanisms or drug actions either due to the complex signaling transduction networks of cells or due to the complicated profile of drug potency and selectivity.

Finding drug's targets is traditionally based on high-throughput *in vitro* assays using recombinant enzymes or protein fragments [2]. The main goal is to characterize the drug's biochemical activity (binding affinities that describe potency and selectivity) and depict them in drug-interaction maps [3]. In most cases, once the target(s) is known, the *in vivo* effect on the signaling pathway is validated by measuring the drug's efficiency to inhibit the activity (usually measured as phosphorylation level [4]) of the downstream protein. However, beyond that measurement, little is know on how the rest of the signaling network is affected. In addition, *in vivo* drug effects can hardly be calculated from *in vitro* assays for several reasons: most kinase inhibitors are promiscuous [5], there is discrepancy between *in vivo* and *in vitro* binding affinities of drugs [6], and there is an additional discrepancy between *in vivo* binding affinities and *in vivo* inhibitor activity for the phosphorylation of downstream signals.

To address drug effects in more physiological conditions, novel genomic and proteomic tools have recently been developed [7]. In the genomic arena, large-scale mRNA analysis (e.g., [8],[9]) enhanced by computational approaches for drug target deconvolution (e.g., [10],[11]) have been developed. Despite the holistic advantages that genomic approaches have to offer, proteomic-based discovery is a step closer to the function of the cell. Towards this goal, affinity chromatography offers a viable strategy for *in-vivo* target identification. This approach utilizes a solid support linked to a bait (usually the drug) to enrich for cellular binding proteins that are identified by mass spectrometry (MS) [12]. However, such experiments usually require large amounts of starting protein, are biased toward more abundant proteins, and result in several hits due to nonspecific interactions [13],[14]. In order to circumvent the non-specific interaction problem, another bait-based strategy uses quantitative MS with “dirty” inhibitors for baits to immobilize the kinome [15],[16]. While this approach significantly reduces the non-specific interaction problem, it also limits the target-searching space to those kinases with the highest affinity to the bait. More recently, quantitative MS-based proteomics using SILAC technology [14] extends the search space to all targets that do not bind covalently to the drug. However, incorporation of the SILAC's isotopes requires 5 population doublings and thus, excludes the application on primary cells with limited replication capabilities. Taken together, all techniques listed above can -in the best case scenario- list the affinities of all targets to the drug but no information is provided whether this binding affinity is capable of inhibiting the transmission of the signal to the downstream protein or how those preferential bindings can collectively affect the signaling network of the cell.

**Figure 1:**
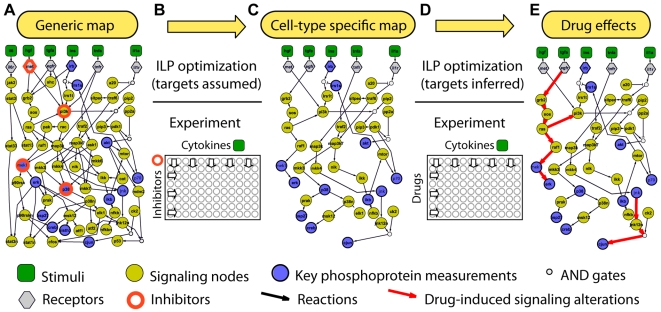
Experimental and computational workflow to assess drug effects. (A) A Boolean generic map is assempled from pathway databases and includes stimuli (green squares), key measured phosphoproteins (brown circles), and the neighboring proteins (yellow circles). (B) Cells are treated with a combination of cytokines and selective inhibitors (red circles) of known effects and an ILP formulation is used to fit the data to the Boolean pathway. (C) A cell-type specific pathway is constructed. (D) Cells are treated with a combination of cytokines and drugs –their effects are assumed unknown- and ILP is used for the second time to fit the drug-induced phosphorylation data. (E) Alterations of the the cell-type specific topology reveals drug effects (red arrows).

Here, we describe a significantly different approach to identify drug effects where drugs are evaluated by the alterations they cause on signaling pathways. Instead of identifying binding partners, we monitor pathway alterations by following key phosphorylation events under several treatments with cytokines. The workflow is presented in Figure 1. On the experimental front, using bead-based multiplexed assays [17], we measure 13 key phosphorylation events under more than 50 different conditions generated by the combinatorial treatment of stimuli and selective inhibitors. Based on the signaling response and an *a-priori* set of possible reactions (i.e. generic pathway), we create a cell-type specific pathway using an efficient optimization formulation known as Integer Linear Programming (ILP). This approach builds upon the Boolean optimization approach proposed in [18]. The ILP is solved using standard commercial software packages to guaranteed global optimality (within a user-defined, numerically small tolerance). To evaluate drug effects, we subject the cells with the same stimuli in the presence of drugs and we tract the alterations of the same key phosphorylation events. Then, we reapply the ILP formulation without *a-priori* assumption of the drug target, and we monitor the changes in the pathway topology with and without drug presence. To demonstrate our approach, we construct a generic map and optimize it to fit the phosphoproteomic data of the transformed hepatocytic cell lines HepG2. Then, we identify the effects of four drugs: the dual EGFR/ErbB-2 inhibitor Lapatinib [19], two potent EGFR kinase inhibitors Erlotinib [20] and Gefitinib [21], and the “dirty” Raf kinase inhibitor Sorafenib [22]. When our method is applied on those 4 drugs we find their main target effect and we also uncover several unknown but equally active off-target effects. In the case of Gefitinib, we find a surprising inhibition of cJUN in the IL1α pathway.

In contrast to previously developed techniques, our method is based on the actual effect on phosphorylation events carefully spread into the signaling network. Theoretically, it can be applied on any type of intracellular perturbations such as ATP-based and allosteric kinase inhibitors, RNAi, shRNA etc. On the computational front, our ILP-based approach performs faster and more efficient than current algorithms for pathway optimization [18] and can identify the main drug effects as well as unknown off-target effects in areas of pathways constrained between the activated receptors and the measured phosphorylated proteins. Our fast and unbiased characterization of modes of drug actions can shed a light into the potential mechanisms drug's efficacy and toxicity.

## Results

### Construction of phosphoproteomic datasets

High-throughput bead-based ELISA-type experiments using xMAP technology (Luminex, Texas, USA) are performed as briefly described in the Materials and Methods section and in [17]. We create two datasets: one for the construction of cell-type specific topology and another for the identification of the mechanisms of drug actions. To do that, HepG2s are stimulated in 10 different ways with combinatorial treatments with a diverse set of 5 ligands (TNFα, IL1α, HGF, INS, TGFα, and no stimuli) and either 4 highly selective inhibitors (PI3K, MEK, p38, cMET, and no inhibitor) or 4 commercial drugs (EGFR inhibitors Lapatinib, Erlotinib and Gefitinib, and the “dirty” inhibitor Sorafenib) (Figure 1b and 1d). For the purpose of this paper, we refer to “inhibitors” as the compounds for which we know the target and we use them in a concentration capable to block ∼95% of the downstream protein. Conversely, we refer to “drugs” as the compounds for which we assume no *a-priori* knowledge of their target. For each combination of cytokine and drug/inhibitor we collect cell lysates at 5 and 25 minutes. The two time points are pooled together in 1∶1 ratio and the mixed lysates are used as an indicator of the “average early signaling response”. For each treatment we measure 13 protein phosphorylations that we consider “key protein activities” (raw data in Figure S1). The key phosphorylation signals (listed in Materials and Methods) are chosen based on the availability of the reagents and quality controls performed at the early phases of the experimental setup [17]. The raw data (arbitrary fluorescent intensities) are normalized to fit logic models as described in [18] using a non-linear transformation that converts raw data into values between 0 and 1 where 1 corresponds to the fully activated state and 0 to no-activation. It has to be noted that logic-transformed data depends on what should be considered “protein activation” (transformed value >0.5), a criterion that is embedded in the transformation function and accounts for signal-to-noise limits, saturation of the detection scheme, and eliminates biases that could have been introduced by the variability of antibody affinities [18].

### Generic pathway assembly and visualization

The generic pathway map is constructed in the neighborhood of the 5 stimuli and the 13 measurements. The ubiquitous presence of conflicting reports on pathway maps and alternative protein names makes this step a highly nontrivial one. We explored several pathway databases including STKE, Pathway Interaction Database, KEGG, Pathway Commons, Ingenuity, and Pathway Studio [23],[24]. Our limited intracellular protein coverage makes impractical the reduction of very large pathway datasets such as those found in Pathway Commons. Here, we create the initial topology from the union of canonical pathways found in Ingenuity (Redwood City, California) with subsequent manual curation.

**Figure 2:**
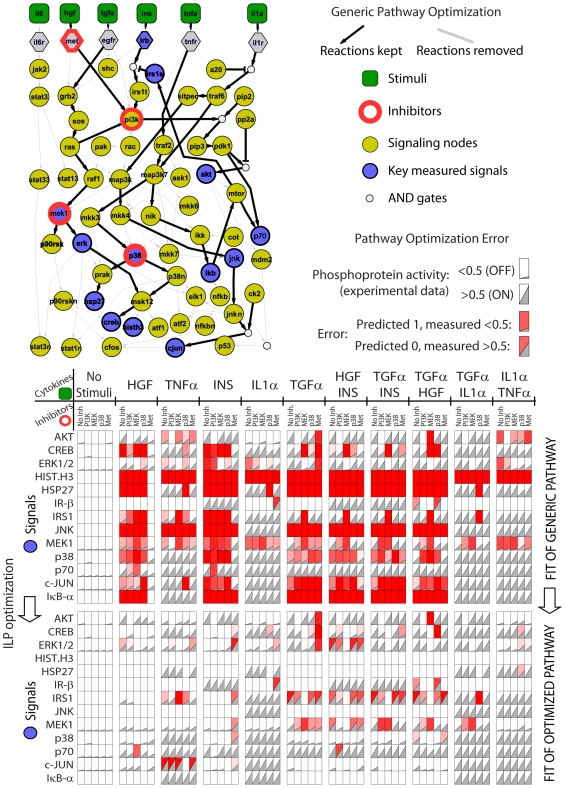
Cell type specific topology using Integer Linear Programming. The ILP algorithm is using a subset of postulated reactions denoted with black and gray arrows in a generic pathway to construct a HepG2 pathway map (black arrows in pathway diagram). Gray triangles show phosphoprotein activation level upon stimuli (columns in top and bottom panels) and inhibitors (subcolumns in top and bottom panels). Red background denotes an error between experimental and pathway-inferred responses. Generic topology can hardly represent the HepG2 signaling responses (red background in top panel) and pathway optimization is critical to obtain a pathway topology that captures HepG2 function (limited red background in bottom panel). Pathways are visualized using Cytoscape [54].

A detailed description of Boolean representation of pathways can be found elsewhere [18], [25]–[29]. In the present manuscript as opposed to [18], the connectivity in our pathway (Figure 2, left panel) is represented with OR gates and only few connections (represented with small black circles in Figure 2) require an AND gate. We are therefore not comparing OR vs. AND gates, but rather assuming our pathways to be ‘causal’ graphs, and since there are a few AND gates we refer to it as Boolean model.

### Construction of cell-type specific pathway via ILP formulation

The formulation for the optimal pathway identification is a 0–1 Integer Linear Program, i.e., an optimization problem with binary variables and linear constraints (see Materials and Methods). The optimizer picks values for the decision variables, such that the logical constraints are satisfied and the objective(s) optimized. The primary objective is to find an optimal pathway, i.e., a pathway that best describes a set of phosphoproteomic data under a given model (e.g. Boolean). A secondary objective is that the pathway is as small as possible, i.e., has as few connections as possible, such that the best-possible fit of the experiments is maintained (see Materials and Methods). It is shown that some of the binary variables can be relaxed to continuous, without changing the feasible set.

The ILP is solved with the state-of-the-art commercial code (CPLEX [30],[31]) that guarantees minimal error between experimental data and the Boolean topology. The goodness of fit (percent error as described in Materials and Methods) was decreased from 36.7% on the generic map to 8.3% on the optimized map (Figure 2). The main source of error is the inability of TGFα to activate the IRS1_s (serine residue of IRS1) (see the red background on the IRS1 row at the bottom panel of Figure 2). This is a result of the infeasibility of the generic pathway to satisfy the activation of IRS1_s in a TGFα/IL1α-dependant but HGF/INS-independent manner: TGFα activation of IRS1_s requires mTOR activation via AKT which the optimization algorithm removes to satisfy the inactivation IRS1_s by INS that shares the same path with TGFα. This example highlights the importance of multi-perturbations to better constrain the optimization formulation.

Figure 2 shows the optimized topology of HepG2s. Our ILP formulation uses two subsequently-imposed objective functions to remove reactions that do not fit the experimental data. During the optimization of the first objective the ILP formulation (A) keeps reactions that lead to phosphorylations of the key proteins and (B) removes reactions that lead to false protein activations. An example of the first case is the Insulin (INS)-induced AKT activation that is maintained via the INS→IRb→IRS1t→PI3K→PIP3→PDK1→AKT path (see INS to AKT path in Figure 2). An example of a removed reaction is the TNFR→PI3K reaction which is removed because there is no TNFα induced AKT activation (see TNFR→PI3K→…→AKT in Figure 2). During the optimization of the secondary objective (see Materials and Methods), several reactions with no evidence of their existence (no downstream measurements, or no stimuli) are removed. In this step, the overall goodness of fit is not improved, but the size of the topology is reduced. To illustrate this case, we add to the initial topology the receptor IL6R but the associated stimulus IL6 is not introduced on the experiments. After the secondary optimization, all downstream reactions of IL6 are removed because no data are present (see reaction arrows downstream for of IL6 in Figure 2). Similarly, all reactions downstream of the bottom-of-the-network key proteins are removed (e.g. CJUN→CFOS reaction in Figure 2). All those reactions might be present in reality and could have been kept if the secondary objective was not present. Here, we apply the secondary objective and follow a network trimming which removes all reactions that might be present in the cell but due to the lack of measured signals or experimental conditions cannot be verified. The resulting network is significantly smaller but contains only elements for which there are solid experimental evidence that explain the topology.

To validate our model, we also examine three scenarios where we remove 20% of our experimental data, and then we try to predict them. Specifically, we create three training datasets, each time by removing all cases where one inhibitor is present (either MEKi, PI3Ki, or p38i) and then we calculate how well our ILP-optimized map can predict each of the inhibitor cases (see Figure S2). For the MEKi, PI3Ki, and p38i scenarios the goodness of fit is 8.22%, 9.46%, 7.05% respectively and our ILP-formulation converges on the same or slightly less optimal solutions compared to the solutions obtained when the whole dataset is used for training (4.47%, 7.76%, and 7.05% respectively) - See Figure S2. Note that the errors given refer only to the subset considered in each case, not the entire dataset. More extensive validations for Boolean-type models on similar phospho-proteomic dataset can also be found in Saez-Rodriguez et al. [18].

### Comparison with genetic algorithm

In order to compare the ILP algorithm with the previously published genetic algorithm (GA) we use the same initial topology and the same normalized dataset [18]. The two algorithms reached almost identical results (see Figure S3). For the ILP, the computational requirements are manageable, in the order of a few seconds (14.3 seconds for this example) on an Quad Core Intel Xeon Processor E5405 (2.00GHz,2X6M L2,1333) running Linux 2.6.25.20 (using only one core). In comparison, the same optimization problem using GA requires approximately 1 hour on a similar power computer. The optimal pathway furnished by the ILP matches all but 98 out of 880 experimental data, as opposed to 110 mismatches in the topology furnished by the GA. It has to be noted that GA does not provide termination criteria, and it is conceivable that after even larger CPU times the GA would have achieved the same fit as the ILP. In contrast the deterministic solution of the ILP guarantees that an optimal fit (not necessarily unique) has been identified within a user-specified tolerance (10^−3^ in our case). In addition to the guaranteed optimal solution, commercial ILP solvers are fast, robust and reliable. Note that open-source ILP solvers also exist, but in our experience are not yet adequate. Note also that for larger network topologies, the differences in CPU time will become even more dramatic, rendering the GA intractable.

The notable differences between the proposed method and the method used in [18] is mainly due to fundamental algorithmic differences: the technology behind deterministic ILP solvers (branch-and-bound, branch-and-cut) is more sophisticated than genetic algorithms, it employs the inherent linearity of the problem, and makes use of the good scalability of linear programs (sub-problems in branch-and-bound tree). In contrast, GA treats the model as a black-box and does not exploit the problem structure. Another point is that herein we used a well-established commercial solver, whereas Saez-Rodriguez et al. [18] used their own implementation of GA. Commercial deterministic ILP solvers, such as CPLEX, rely on several decades of research and development, and have extremely powerful features such as pre-processors and node selection heuristics. Thus, they typically become the default choice for ILPs.

### Identifying drug effects via drug-induced topology alterations

For the identification of the drug effects we make use of the second dataset in HepG2s where drugs are applied together with the same set of ligands. In this case, the ILP formulation is being used with the HepG2 specific topology (topology obtained from the previous step) and not the generic map. We also do not impose inhibitor constrains the way we do for pathway optimization (e.g., PI3K inhibitor blocks the signal downstream of PI3K) but we let the optimization algorithm decide which reaction(s) should be removed in order to fit the drug-induced data.

**Figure 3:**
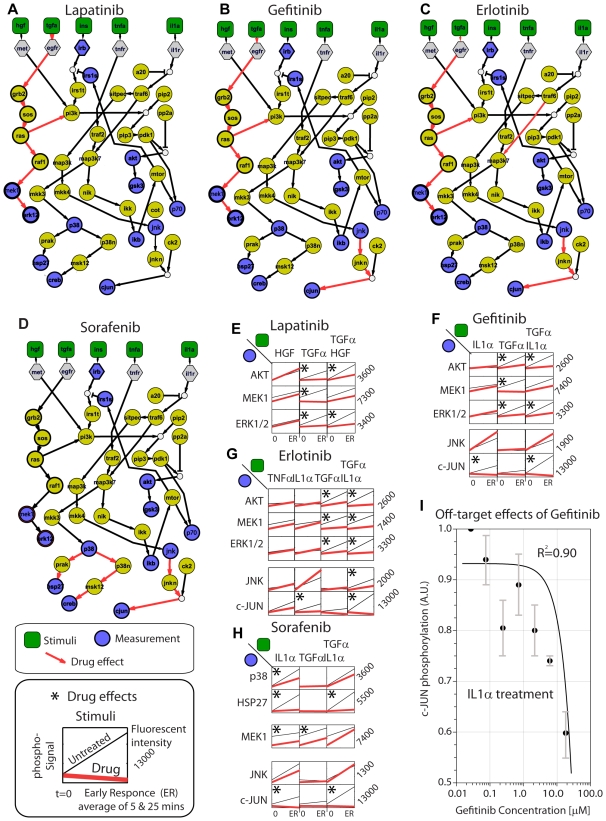
Drug-induced pathway alterations. (A–D) Red arrows denote drug effects, i.e., reactions that are removed from the HepG2 topology by the ILP algorithm in order to fit the drug-altered phosphoprotein dataset. (E–H) Raw data that correspond to drug effects. Lines indicates the signal between 0 minutes (untreated) and “early response” (average signal of 5 and 25 minutes post stimuli). (I) Off-target effect of Gefitinib. Dose response curve shows that the EGFR inhibitor reduces cJUN activation upon IL1α treatment. R^2^ corresponds to linear fit.

The effect of Lapatinib (Figure 3a), the most selective and specific EGFR inhibitor [32], is the complete removal of the downstream reactions of the TGFα branch: TGFα→GRB2→SOS→RAS→PI3K and RAS→RAF1→MEK1/2→ERK1/2. This resulted from the fact that Lapatinib blocks the TGFα induced MEK1/2, ERK1/2, and AKT phosphosignals (Figure 3e). Note that the PI3K→…→AKT branch is not removed because it is being used by the HGF and INS path for the activation of AKT that cannot be blocked by Lapatinib (Figure 3e).

Gefitinib, an EGFR tyrosine kinase inhibitor, alters the topology in a very similar pattern as Lapatinib, but, interestingly enough, it also results in the removal of the JNK→c-JUN branch (Figure 3b). Closer examination of the raw data (Figure 3f) shows a potent inhibition of IL1α- and (IL1α+TGFα)-induced cJUN activity upon Gefitinib treatment. To follow up this interesting off-target effect, we did a dose-response experiment where Gefitinib shows that it can reduce the activation of cJUN signal induced by the IL1α stimuli (Figure 3i). We believe that the inhibition of cJUN is not due to the binding of Gefitinib in the upstream molecule JNK but a collective effect of signaling inhibitions in several species that take part in the path between IL1α and cJUN. For this reason, a fitting with a typical dose response curve has been avoided and a simple linear equation has been used instead (Figure 3i). Erlotinib, another EGFR inhibitor, has the same effects as Gefitinib (Figure 3c) but at the same time shows an effect in the TRAF6→MAP3k7 reaction. This effects is probably because IκB-α is inhibited in an IL1α -dependent but TNFα-independent manner (see IκB-α signals upon IL1α and TNFα stimuli in Figure S1); the only way for the ILP to satisfy this behavior is to remove the transmission of signal before the merging of TNFα and IL1α paths which can be done through the TRAF6→MAP3K reaction.

The “dirty” Raf inhibitor Sorafenib shows a very different profile: it also blocks the JNK→c-JUN branch (Figure 3d) and in addition affects the p38 path (see complete HSP27 inhibition upon IL1α treatment in Figure 3h). An interesting observation is that network optimization does not remove the RAF→ERK1/2 reaction despite the fact that RAF is the main target of Sorafenib. Close inspection of the data shows that Sorafenib reduces but does not block the MEK1 phosphorylation (see MEK phosphorylation in Figure 3h). This is in agreement with previous published results where Sorafenib does not inhibit activation of the RAF/MEK/ERK pathway in all human tumor cell lines [33] a finding that highlights the importance of *in-vivo* assays for the quantification of drug effects.

## Discussion

In this article, we present an unbiased phosphoproteomic-based approach and an optimization formulation to construct cell-type specific pathways and to identify drug effects on those pathways. For the pathway construction, we track 13 key phopshorylation signals in 55 different conditions generated by the combinatorial treatment of stimuli and inhibitors. Using Integer Linear Programming (ILP) for pathway optimization we take a generic network of 74 proteins and 105 reactions and construct a cell-type specific network of 49 proteins and 44 reactions that spans between the 5 stimuli and the 13 measured phosphorylated proteins. In this network, we monitor 4 cases of drug-induced pathway alterations using a similar computational scheme.

In comparison to all other protein-based target identification approaches, our method is not based on measurements of drug affinities either by *in vitro* or *in vivo* assays. Instead, we use an “operative” signaling network and rely on key phosphorylation events and *a-priori* knowledge of possible connections to reveal the topology and monitor its alterations under the presence of the drug. Thus, our method is expandable to any type of intracellular perturbations such as ATP-based and allosteric inhibitors, RNAi, shRNA etc. Since no bait or MS is required, we have simple ELISA-type experimental procedure with minimal requirements of cell starting protein (∼30,000 cells per condition), without affinity immobilizations, protein fractionations, or carefully optimized wash conditions. With our current semi-automated procedures in our lab (robotic liquid handlers), we can achieve total experimental and computational time for a similar size experiment in less than a week. On the other side, our approach can only detect signaling alterations in topologies bounded between the applied stimuli and the measured phosphorylated proteins and it misses off-target effects outside the constructed network. The expansion of the constructed network depends primarily on three factors: highly curated generic topology, multiplex assay availability for “key” phosphorylation measurements, and experimental cost. We believe that the explosive growth of multiplexed phosphoproteomic assays, the rapid reduction of the cost per datapoint, and the significant improvement in quality of several pathways databases will significantly increase the searching space for drug effects using our proposed methodology. However, our search space will always be significantly smaller compared to whole-genome based approaches [8]–[11] because it requires (a) the input of a generic pathway which is available only in well-studied pathways and (b) good quality antibodies for the detection scheme. By merging our phosphoproteomic method with genome-wide screening techniques, we might be able to combine the strengths of both approaches and increase the searching space for off-target drug effects.

An important aspect of the current approach is the construction of pathway maps. Pathway construction is a major endeavor in biology and a variety of experimental [34]–[38] and computational approaches that span from data-driven methodologies (e.g., statistical, unsupervised machine learning) to topology-based methods (e.g., kinetic models based on ordinary differential equations-ODEs) [17], [35], [38]–[41] have been developed. Our approach, which is based on Boolean (logical) modeling [26]–[28],[42], represents a simplified topology-based method. Compared to ODE-based methods, a logic model has limited abilities to model kinetic behavior [25] (especially when modeling feedback loops in single-step logic models) or even to model the protein activity in a continuous fashion. On the flip side, logic models do not require parameter estimation (sometimes ill-defined from lack of experimental data) and thus can be applied for the simulation of large topologies. A refinement of the model formalism into multistep logic [28], fuzzy logic [43], or ODE-based logic systems [44] may provide a more precise simulation of the activity and time-dependency of the signaling network. Taking into account the current limitations of experimental assays (throughput, sensitivity, reliability, cost) we believe that Boolean modeling is the method of choice with high predictive power when large topologies are studied.

Optimizing pathway topologies is a relatively new approach for the construction of cell-type specific pathways. Using Boolean topology and Genetic Algorithm (GA) for an optimization scheme, Saez-Rodriguez et al. [18] are able to fit a generic map to cell-type specific map from phosphoprotein data. Here we present an alternative method of optimal pathway identification based on ILP. Compared to GA, our algorithm gives guaranteed globally optimized map (the solution identified is guaranteed to be no worse than 0.001 than any other possible solution). Additionally, the computational cost has cut down dramatically and allows pathway optimization with ∼70 species to be performed on a desktop computer in a matter of few seconds. Due to minimal computational requirements ILP can be used for the construction of large pathways (assuming that experimental capabilities can by matched) and for the exploration of alternative reactions beyond the generic topology to further improve the optimal fit. However, several factors should be addressed before expanding our formulation to larger topologies. Although our formulation is able to identify a globally optimal solution, additional optimal solutions might exist [18] in the same generic network and further more solutions might arise when the optimization formulation is relaxed. Larger and more interconnected networks increase the number of solutions that are equally (or near equally) optimal. A possible way to circumvent this problem is to reduce our network using techniques that have been described previously in graph theory or in [18]. Being aware of those limitations in the present manuscript we described a “simple” and not highly interconnected network in order to minimize redundancy of solutions. To address the issue of finding a both unique and optimal solution we are currently working on two complementary approaches: (a) instructing the ILP solver to furnish a pool of near-optimal solutions and (b) devising “clever stimulations” by taking into account experimental limitations (i.e., combination of inhibitors, stimuli, and key protein measurements) that maximally constrains the optimization scheme and gives smaller number of unique solutions.

When applied in HepG2s, our approach identifies both known and unanticipated results. As a positive control, it removes the TGFα branch upon EGRF drug treatments. Another easily understandable effect is Sorafenib's inhibition of the pathway downstream of p38 which can be explained by the drug's target affinity to p38α and p38β [32],[45]. A surprising effect is the removal of the JNK→cJUN reaction under the influence 3 out of 4 cancer drugs Erlotinib, Gefitinib and Sorafenib. Interestingly, kinase profiles of those drugs [32] shows no medium or high affinity for the directly upstream JNK1/2 kinases. Despite that, Gefitinib shows a significant reduction of the cJUN activity upon IL1α treatment. A possible explanation is that the signaling propagation can collectively be attenuated from the low or medium off-target inhibitions of several kinases upstream of JNK and cJUN. This also might explain the inhibition curve in Figure 3i, where Gefitinib inhibition of cJUN activation does not follow a typical dose-response curve. In this context, sensitivity analysis in ODE-based pathway models [46] have shown that slight changes of reaction constants can have significant attenuations on protein activities several steps downstream the network and thus inhibitory curves cannot be simulated by simplified dose-response models. Our findings also highlight a unique feature of our approach: we find effects of drug's promiscuity that cannot be identified by the direct binding of the drug to the upstream target but are the result of a collective effect of drug's interactions with several upstream molecules. Bait-based analysis cannot reveal those effects since there is no binding involved between the drug and the protein.

Understanding the interplay between *cell function* and *drug action* is a major endeavor in the pharmaceutical industry. Here, we provided a methodology to construct cell type specific maps and identify drug effects on those maps. Our ILP formulation was able to build the best possible topology from a set of *a-priori* determined reactions and choose those, where their presence is confirmed from high throughput phosphoprotein data. Since phosphorylation events are the ultimate reporters of protein/drug function the use of high-throughput phosphoproteomic datasets gave an advantage in data quality for modeling signaling network. We believe our approach complements standard biochemical drug profiling assays and sheds new light into the discovery of possible mechanisms for drug's efficacy and toxicity.

## Materials and Methods

### Experimental procedure: phosphoprotein dataset

HepG2 cells were purchased from ATCC (Manassas, VA), and seeded on 96-well plates coated with collagen type I-coated (BD Biosciences, Franklin Lakes, NJ) at 30,000 cells/well in DME medium containing 10% Fetal Bovine Serum (FBS). The following morning, cells were starved for 4 hours and treated with inhibitors and/or drugs. Kinase inhibitors were used at concentrations sufficient to inhibit at least 95% the phosphorylation of the nominal target as determined by dose-response assays (presented in [17]). AKT was chosen as the nominal target for Lapatinib, Erlotinib, and Gefitinib. The following saturated concentrations were used: p38 (PHA818637, 20 nM), MEK (PD325901, 100 nM) and cMET (JNJ38877605, 1µM), PI3K (PI-103, 10 µM), Lapatinib at 3uM [47], Erlotinib at 1 uM [47], Gefitinib at 3uM [47], and Sorafenib at 3 uM (based on its inhibitory activity on ERK1/2 phosphorylation [33]). Following incubation for 45 minutes with inhibitors and/or drugs cells were treated with saturated levels of 5 ligands: Tumor Necrosis Factor alpha (TNFα) at 100ng/ml, Interleukin 1 alpha (IL1α) at 10ng/ml, Insulin (INS) at 2uM, Transforming Growth Factor (TGFα) at 100ng/ml, and Hepatocytes Growth Factor (HGF) at 100 ng/ml. Each ligand was added alone or in pairs and cell lysates were collected at 0, 5, and 25 minutes following the cytokine stimulation. The 5 and 25 minutes lysates were mixed together in 1∶1 ratio and the mixed lysate was measured as an indicator of the “average early signaling response”. The 5 and 25 minute time points were identified in a preliminary experiment as the optimal time points that maximally captured early phosphorylation activities [17].

A major improvement in the present dataset as compared to [17] was the “in-vitro” averaging of the signals from 5 and 25 minutes rather than “in-silico” averaging (i.e., first both time points are measured, then we take the average). Three are the main advantages using such approach: 1) two signals are used instead of one and thus very early signalling responses can be captured, 2) the experimental cost is reduced by 50% (or more for averaging multiple time points), and 3) we achieved the averaging of some signals that could not be measured independently because their “active” state is reaching the saturation limits of our measuring instrument.

From each lysate we measured 13 phosphorylation activities that we considered “key phosphorylation events” using a Luminex 200 system (Luminex Corp, Austin, TX). The 13-plex phospho-protein bead set from Bio-Rad was used to assay p70S6K (Thr421/Ser424), CREB (Ser133), p38 (Thr180/Tyr182), MEK1 (Ser217/Ser221), JNK (Thr183/Tyr185), HSP27 (Ser78), ERK1/2 (Thr202/Tyr204, Thr185/Tyr187), c-JUN (Ser63), IRS-1 (Ser636/Ser639), IκB-α (Ser32/Ser36), Histone H3 (Ser10), Akt (Ser473), and IR-β (Tyr1146). Data were normalized and plotted using with DataRail [48]. For the construction of the dose response curve in Figure 3i, HepG2 were starved for 4 hours and then incubated with Gefitinib (from 20uM down to 27nM – 3 fold dilution) for 45 minutes followed by incubation with IL1α at 10ng/ml final concentration for 30 minutes. Duplicate lysates were analyzed using the c-JUN (Ser63) beads in the Luminex 200 system.

### Computational procedure: ILP formulation

Here, we describe how the Boolean model described in [18] can be reformulated as an ILP. Note that such a transformation was recently performed for a different problem, namely the satisfiability, by [49]. A pathway is defined as a set of reactions 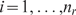 and species 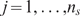. Each reaction has three corresponding index sets, namely the index set of signaling molecules 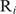, inhibitors 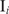, and “products” 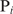 (“product” can also correspond to the phosphorylation level of the protein). These sets are all subsets of the species index set (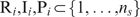). Typically, these subsets have very small cardinality (few species), e.g., 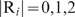; 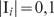; 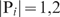; 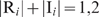. A reaction takes place if and only if all reagents and no inhibitors are present. If a reaction takes place, all products are formed. Note that reactions without products as well as reactions with neither reagents nor inhibitors will be excluded here.

While typically the set of species is known, the set of reactions is not known. Rather, only a superset of potential reactions is postulated. The goal of the proposed formulation is to find an optimal (in some sense) set of reactions out of such a superset. To that extent binary variables 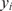 are introduced, indicating if a reaction is possible or not (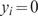 connection not present, 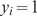 connection present).

A set of experiments is performed, indexed by the superscript 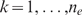. In each experiment a subset of species is introduced to the system and another subset is excluded from the system. These are summarized by the index sets 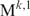 and 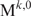 respectively (two for each experiment). In the proposed formulation, constants are introduced for all such species, respectively 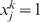 and 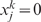. In the following it will be assumed that these species do not appear as products in any reaction; this assumption is not limiting, since in the experiments performed only extracellular species and inhibitors are manipulated. In the experiments a third subset of the species is measured (index set 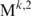) and for the remaining species no information is available. In the proposed formulation for each of the experiments and each such species a binary decision variable 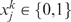 is introduced indicating if the species 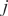 is present (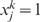) or not (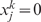) in the experiment 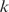 according to the model predictions. It is proved that in the absence of loops, 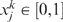 can be used for species that are not input species (see Text S1). This has some computational advantages.

The last group of variables 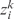 introduced indicate if reaction 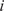 will take place (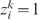) or not (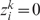) in the experiment 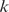 according to the model predictions. It is proved that a real variable 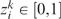 can be used equivalently (see Text S1). This reformulation has some computational advantages.

For the case that a species is measured, the measurement is defined as 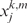. For Boolean measurements 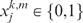; otherwise 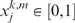 (assuming a scaling as afforementioned). The primary objective function is formed aiming to minimize the weighted error between model predictions and measurements 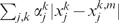. The absolute value is reformulated as 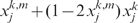. It can be easily verified that for binary 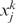 and for 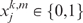 this reformulation is valid:

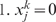
:
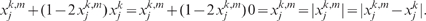

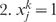
:
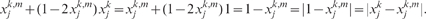

Note also that alternative norms, such as least-squares errors, could be also used. The resulting optimization problem would still be an ILP, since the objective function involves only integer variables. For instance for the least-square error objective function the following linear reformulation is valid:
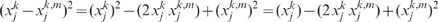



The secondary objective is to minimize the weighted number of possible reactions 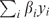. In multiobjective optimization typically the concept of *Pareto-optimal* or *noninferior solution* is introduced, i.e., a set of decision variable values, such that if one tries to improve one objective, another will be degraded [50]. The set of Pareto points forms the Pareto-optimal curve. Here, however, the primary objective is considered much more important than the secondary objective. Therefore, a single Pareto-optimal point is obtained, by first minimizing the primary objective and then the secondary objective by requiring that the former (more important) objectives are not worsened, see also [51]–[53].

The ILP proposed can be summarized as:
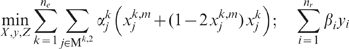
(1)

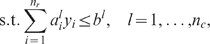
(2)

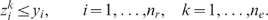
(3)

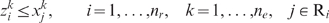
(4)

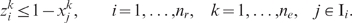
(5)

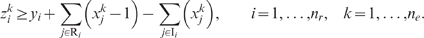
(6)

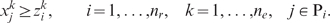
(7)

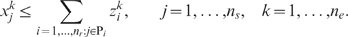
(8)

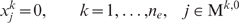
(9)

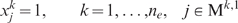
(10)

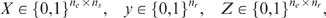
(11)where the objectives are separated by a semi-colon. Note that for the elements of the matrices 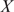 and 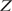, the row index (experiment) is indicated as superscript, and the column index (species and reactions respectively) is indicated as subscript.

In formulation (1)–(11) for the manipulated species binary decision variables along with the constraints (9) and (10) are introduced. This simplifies notation. In the implementation, these variables are replaced by constants. Alternatively the preprocessor of the optimization solver can be used to exclude these trivial variables.

In the following the reasoning for the formulation is given. The first set of constraints, i.e., (2) allow the modeler to limit the combinations of connectivities considered. For instance, suppose that two reagents 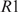, 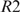 form a product 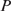, but it is not known if both reagents (AND) or either (OR) are required. This can be modeled as three potential reactions
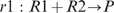


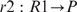


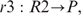
with the additional constraint that 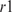 excludes 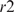 and 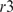, which can be modeled as two linear inequalities:
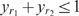


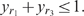
The constraints (3) indicate that a reaction can only take place if it is possible (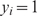). This can be seen easily, since 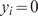, gives 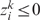 and together with 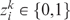 we obtain 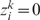. Similarly, the constraints (4) and (5) ensure respectively that a reaction can only take place if all reagents and no inhibitors are present. If for instance a reagent is absent, 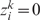 is enforced, and the other constraints are redundant. On the other hand, the constraints (6) enforce that if a reaction is possible, all reagents are present, and no inhibitors are present, then the reaction will take place (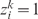).

The constraints (7) ensure that a species will be formed if some reaction in which it is a product occurs. Note that multiple reactions can give the same species; mathematically this will result in redundant constraints. In contrast, the constraints (8) enforce that a species will not be present if all reactions in which it appears as a product do not occur. Recall that manipulated species are not considered as products in reactions. Note also, that it would be possible to combine the constraints (7) into a single constraint for each species, e.g.,
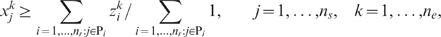
but this would result in weaker LP-relaxations. Also the reformulation of 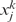 to 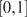 would no longer be exact.

In the present study, our ILP formulation was utilized in two different circumstances. For the creation of the cell-type specific pathway using combinations of inhibitors and stimuli our ILP formulation included 27887 constraints and 9732 variables. For each drug case, where the reduced and optimized pathway was utilized, we had 2477 constraints and 947 variables.

### Computational procedure: goodness of fit

For the goodness of fit, we calculated the percentage error as:
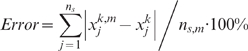



Note that for binary 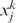 and 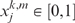 the percentage error cannot be 0% even when there is no mismatch between model and experiment data. Another way to quantify the goodness of fit is by counting the number of mismatches: the cases where the rounded experimental value (0 or 1) is not the same with the computational value, or in other words, when experimental – computational error is more than 0.5.

## Supporting Information

Figure S1 Raw data for the construction of the cell-type specific map and the evaluation of the drug effects. The signals in the Y-axis correspond to the measurements of the phosphorylated residues listed in Materials and Methods. Each column corresponds to cytokine or cytokine mix and each sub-column to the presence of an inhibitor or drug. The numbers to the left are the maximum values across all treatments measured as arbitrary fluorescent intensities.
(0.52 MB PDF)

Figure S2 Model Validation. The first panel shows the optimization results when the full dataset (shown in Figure 2) has been used as training dataset. To validate our model, we created three subsets, in which 20% of our experimental cases are removed that correspond to the treatments with PI3K inhibitor (2nd panel), MEK inhibitor (3rd panel), and p38 inhibitor (bottom panel), and we trained our model against them. The data left out is then used as test dataset for prediction (see highlighted strips in each panel). The error of prediction of the test subsets (error = goodness of fit as describes in Materials and Methods) is shown on the right of each panel.
(0.91 MB PDF)

Figure S3 Comparison between genetic algorithm and ILP. Both algorithms performed well and achieved very similar solutions. Red background denotes inconsistency between predicted values and experimental data: ILP matched all but 98 out of 880 experimental data, as opposed to 110 mismatches in the topology furnished by the GA. The computational time for ILP was 14.3 sec as opposed to 1approximately one hour for GA.
(0.63 MB PDF)

Text S1 Equivalent reformulation as MILP
(0.03 MB PDF)
