# Supplementary figures and images for: Identifying Drug Effects via Pathway Alterations using an Integer Linear Programming Optimization Formulation on Phosphoproteomic Data
Source: PLoS Comput Biol. 2009 Dec 4;5(12):e1000591. doi: 10.1371/journal.pcbi.1000591 (PMC2776985; doi:10.1371/journal.pcbi.1000591)

DATASET WITH INHIBITORS

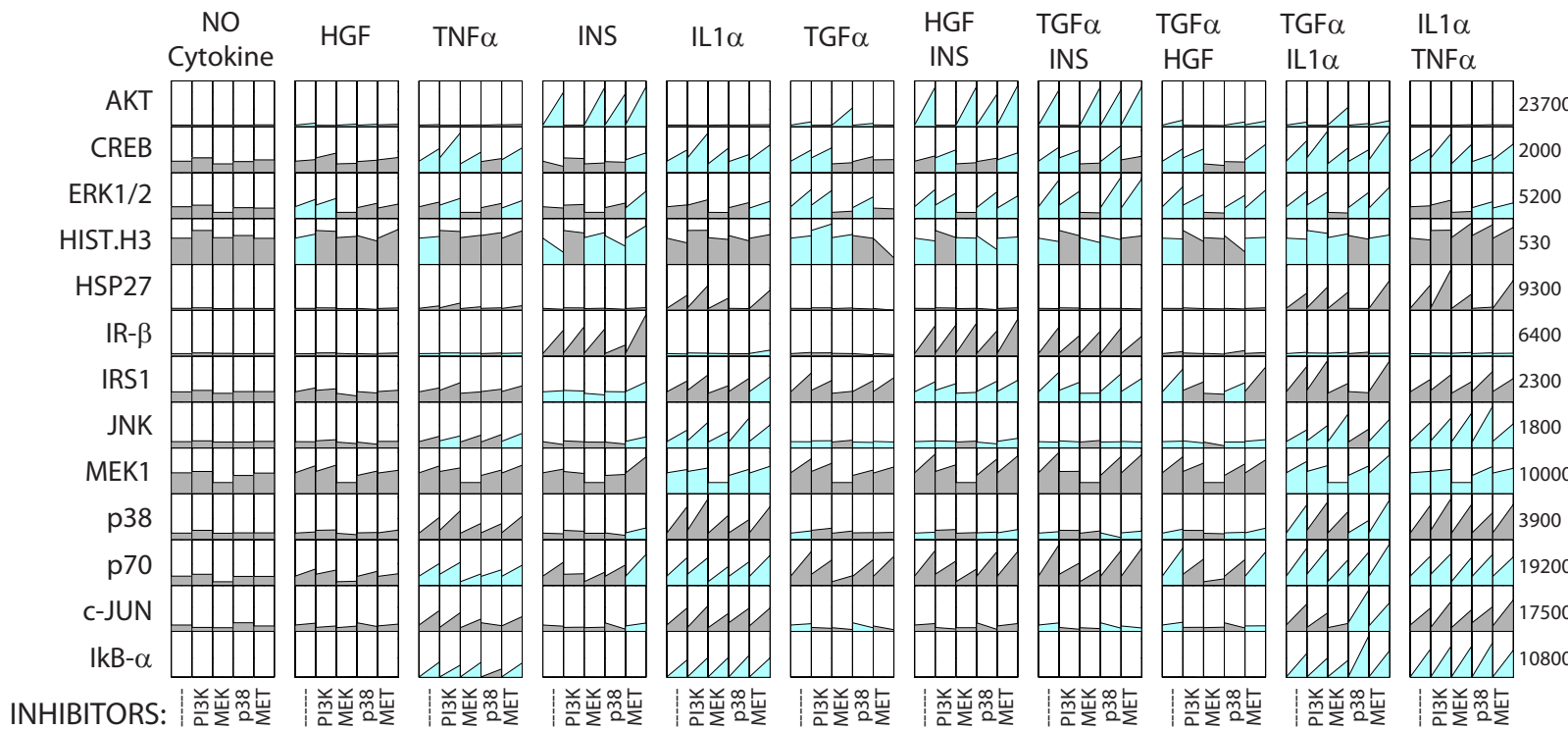

DATASET WITH DRUGS

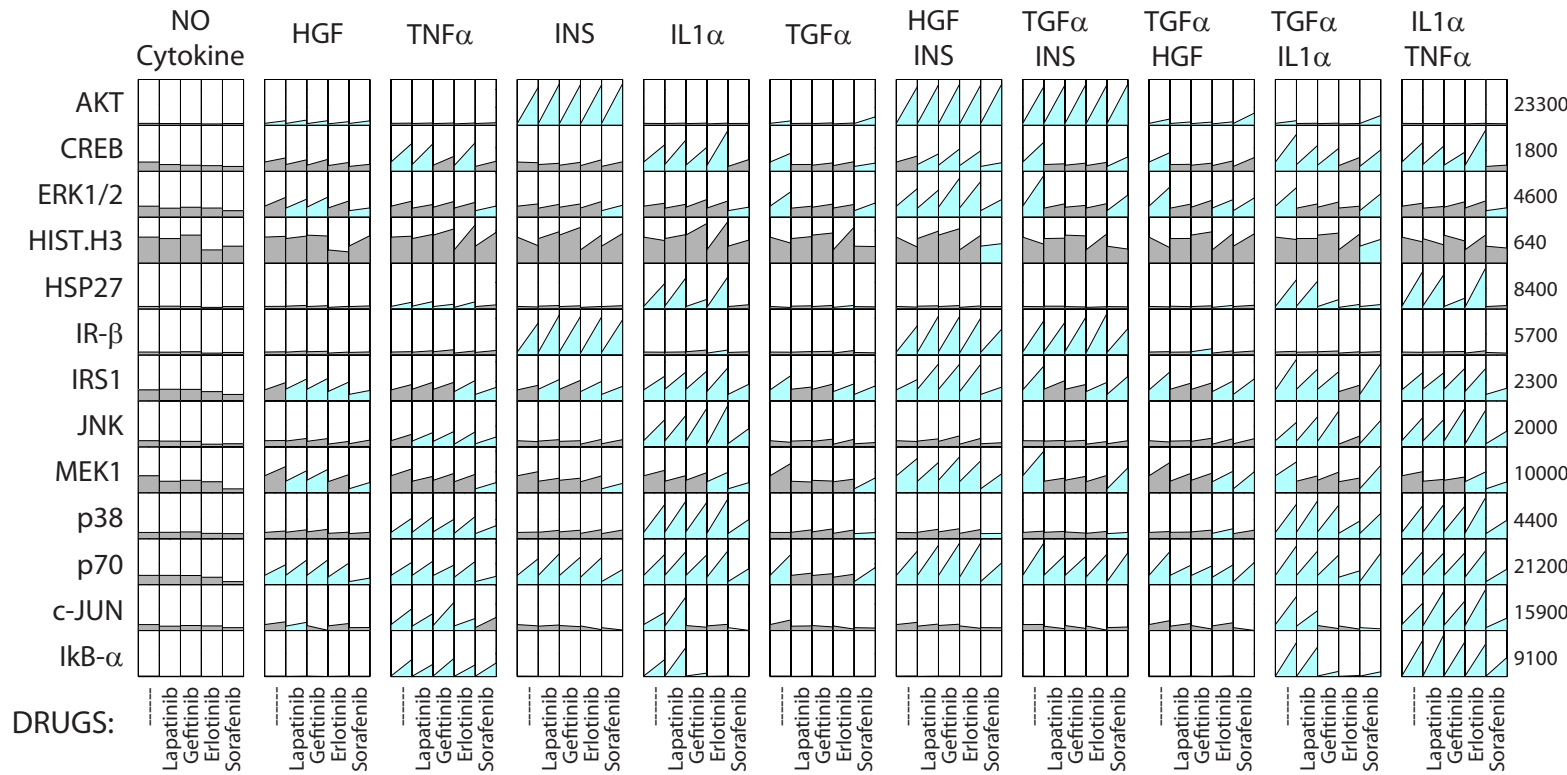

Supplement: Figure S1 — Raw data for the construction of the cell-type specific map and the evaluation of the drug effects. The signals in the Y-axis correspond to the measurements of the phosphorylated residues listed in Materials and Methods. Each column corresponds to cytokine or cytokine mix and each sub-column to the presence of an inhibitor or drug. The numbers to the left are the maximum values across all treatments measured as arbitrary fluorescent intensities. (0.52 MB PDF) [file pcbi.1000591.s001.pdf]

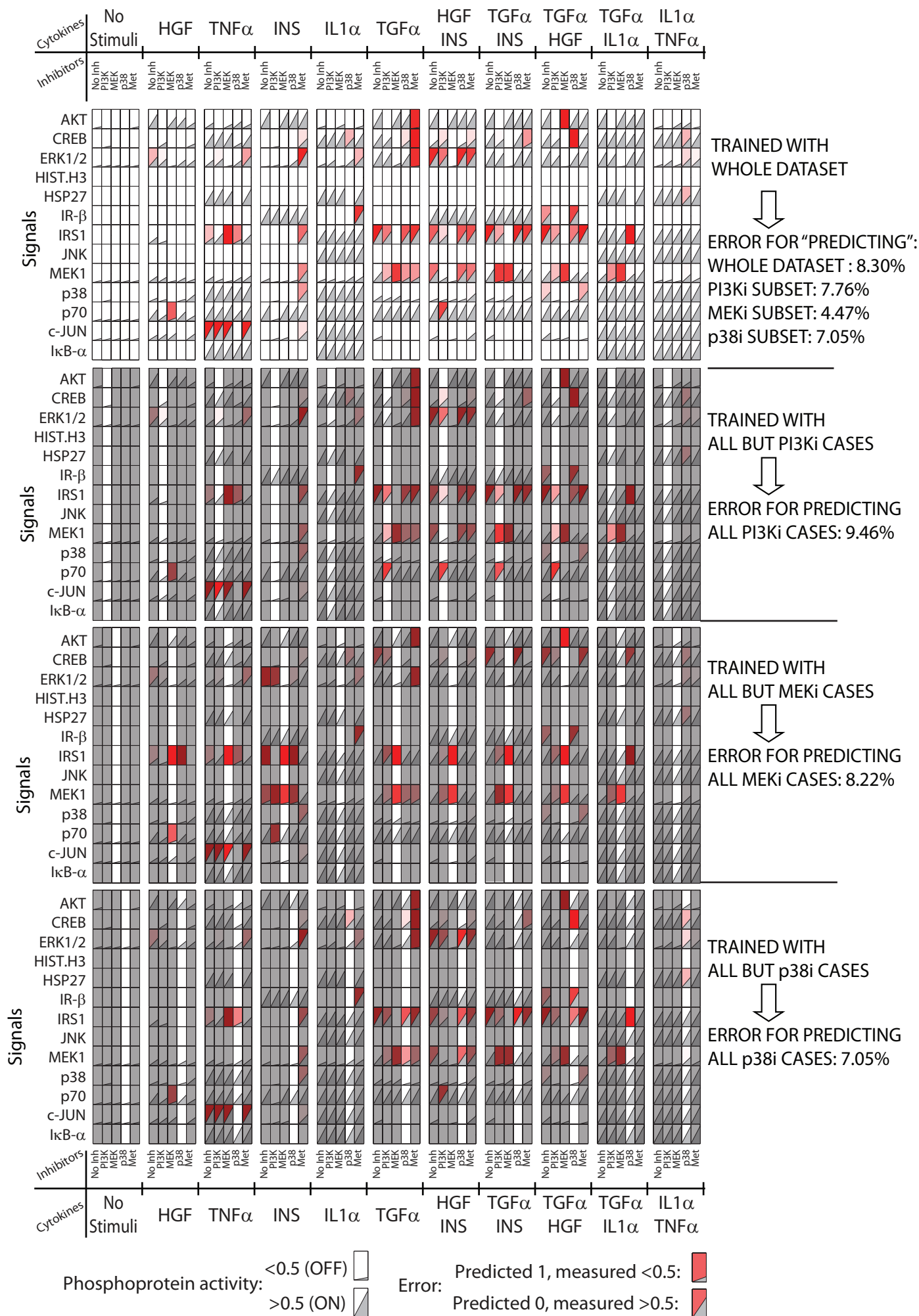

Supplement: Figure S2 — Model Validation. The first panel shows the optimization results when the full dataset (shown in Figure 2) has been used as training dataset. To validate our model, we created three subsets, in which 20% of our experimental cases are removed that correspond to the treatments with PI3K inhibitor (2nd panel), MEK inhibitor (3rd panel), and p38 inhibitor (bottom panel), and we trained our model against them. The data left out is then used as test dataset for prediction (see highlighted strips in each panel). The error of prediction of the test subsets (error = goodness of fit as describes in Materials and Methods) is shown on the right of each panel. (0.91 MB PDF) [file pcbi.1000591.s002.pdf]
