# Supplementary material for: Identifying Drug Effects via Pathway Alterations using an Integer Linear Programming Optimization Formulation on Phosphoproteomic Data
Source: PLoS Comput Biol. 2009 Dec 4;5(12):e1000591. doi: 10.1371/journal.pcbi.1000591 (PMC2776985; doi:10.1371/journal.pcbi.1000591)
Supplement: Figure S3 — Comparison between genetic algorithm and ILP. Both algorithms performed well and achieved very similar solutions. Red background denotes inconsistency between predicted values and experimental data: ILP matched all but 98 out of 880 experimental data, as opposed to 110 mismatches in the topology furnished by the GA. The computational time for ILP was 14.3 sec as opposed to 1approximately one hour for GA. (0.63 MB PDF) [file pcbi.1000591.s003.pdf]

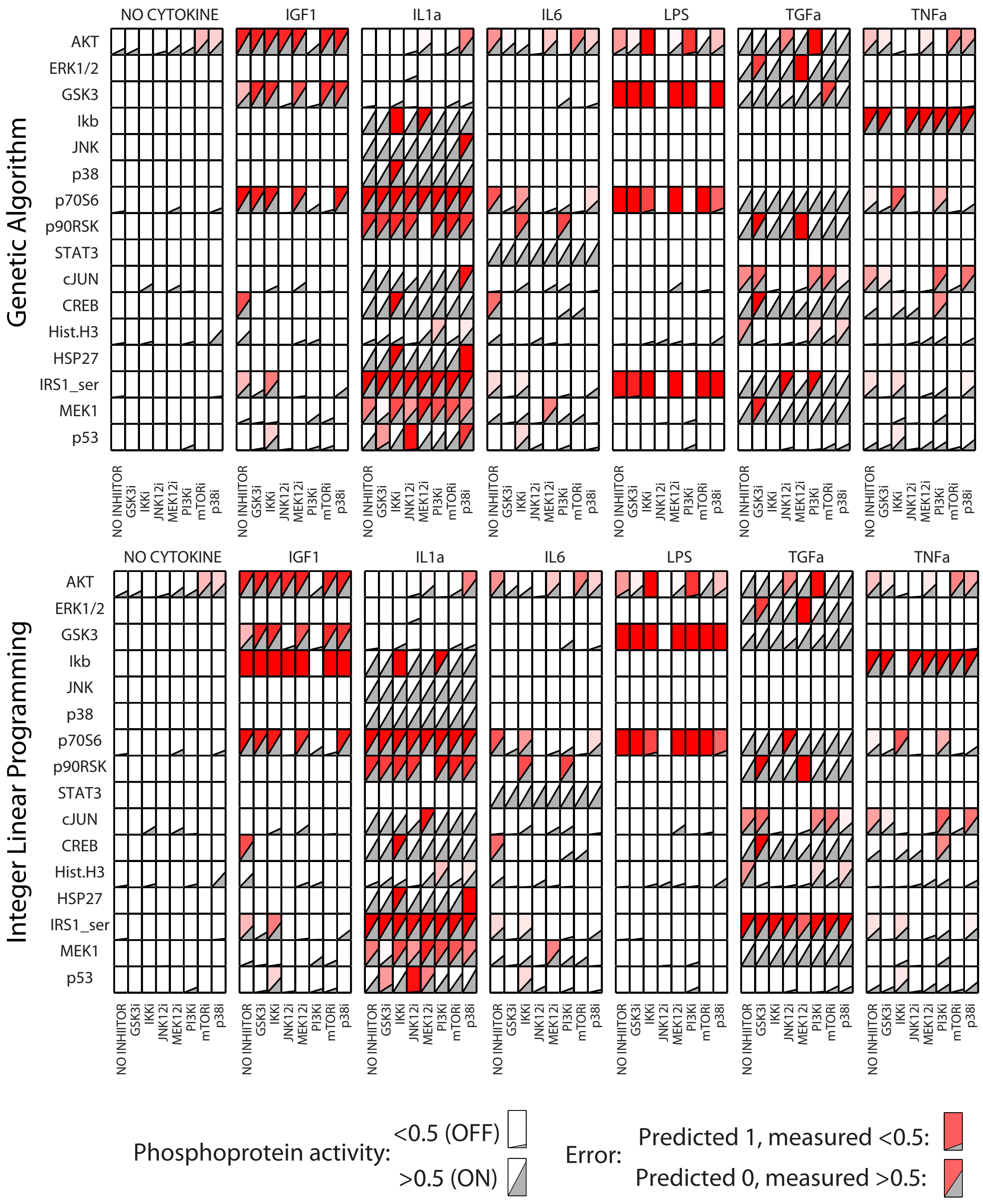
Genetic Algorithm
Integer Linear Programming
NO CYTOKINE
IGF1
IL1a
IL6
LPS
TGFa
TNFa
AKT
ERK1/2
GSK3
Ikb
JNK
p38
p70S6
p90RSK
STAT3
cJUN
CREB
Hist.H3
HSP27
IRS1_ser
MEK1
p53
NO INHIITOR
GSK3i
IKKi
JNK12i
MEK12i
PI3Ki
mTORi
p38i
Phosphoprotein activity:
<0.5 (OFF)
>0.5 (ON)
Error:
Predicted 1, measured <0.5:
Predicted 0, measured >0.5:
