# Supplementary material for: Identifying Drug Effects via Pathway Alterations using an Integer Linear Programming Optimization Formulation on Phosphoproteomic Data
Source: PLoS Comput Biol. 2009 Dec 4;5(12):e1000591. doi: 10.1371/journal.pcbi.1000591 (PMC2776985; doi:10.1371/journal.pcbi.1000591)
Supplement: Text S1 — Equivalent reformulation as MILP (0.03 MB PDF) [file pcbi.1000591.s004.pdf]

Supplemental material S1: Equivalent reformulation as MILP

Recall that the ILP proposed is:

$$\min_{X,y,Z} \sum_{k=1}^{n_e} \sum_{j \in \mathrm{M}^{k,2}} \alpha_j^k \left(x_j^{k,m} + (1-2x_j^{k,m})x_j^k\right); \quad \sum_{i=1}^{n_r} \beta_i y_i \tag{1}$$

$$\text{s.t.} \sum_{i=1}^{n_r} a_i^l y_i \le b^l, \quad l = 1,\ldots,n_c, \tag{2}$$

$$z_i^k \le y_i, \qquad i = 1,\ldots,n_r, \quad k = 1,\ldots,n_e. \tag{3}$$

$$z_i^k \le x_j^k, \qquad i = 1,\ldots,n_r, \quad k = 1,\ldots,n_e, \quad j \in \mathrm{R}_i \tag{4}$$

$$z_i^k \le 1 - x_j^k, \qquad i = 1,\ldots,n_r, \quad k = 1,\ldots,n_e, \quad j \in \mathrm{I}_i. \tag{5}$$

$$z_i^k \ge y_i + \sum_{j \in \mathrm{R}_i} \left(x_j^k - 1\right) - \sum_{j \in \mathrm{I}_i} \left(x_j^k\right), \qquad i = 1,\ldots,n_r, \quad k = 1,\ldots,n_e. \tag{6}$$

$$x_j^k \ge z_i^k, \qquad i = 1,\ldots,n_r, \quad k = 1,\ldots,n_e, \quad j \in \mathrm{P}_i. \tag{7}$$

$$x_j^k \le \sum_{i=1,\ldots,n_r : j \in \mathrm{P}_i} z_i^k, \qquad j = 1,\ldots,n_s, \quad k = 1,\ldots,n_e. \tag{8}$$

$$x_j^k = 0, \qquad k = 1,\ldots,n_e, \quad j \in \mathrm{M}^{k,0} \tag{9}$$

$$x_j^k = 1, \qquad k = 1,\ldots,n_e, \quad j \in \mathrm{M}^{k,1} \tag{10}$$

$$X \in \{0,1\}^{n_e \times n_s}, \quad y \in \{0,1\}^{n_r}, \quad Z \in \{0,1\}^{n_e \times n_r}, \tag{11}$$

Relaxation of Z

We will argue that relaxing the Z variables from binary to continuous gives an exact reformulation. It suffices to show that constraints (3)-(8) together with $X \in \{0,1\}^{n_e \times n_s}$ and $y \in \{0,1\}^{n_r}$ imply $Z \in \{0,1\}^{n_e \times n_r}$.

Theorem 1 *Replacing $Z \in \{0,1\}^{n_e \times n_r}$ by $Z \in [0,1]^{n_e \times n_r}$ is an exact reformulation, in the sense that any feasible point in the new program is also feasible in the original program.*

Note that Theorem 1 is a special case of Theorem 2. Nevertheless it is given separately, because it does not require the assumption of acyclical graphs. Moreover, its proof is much simpler, and is used in the proof of Theorem 2.

Proof. Take any $X \in \{0,1\}^{n_e \times n_s}$, $y \in \{0,1\}^{n_r}$ and $Z \in [0,1]^{n_e \times n_r}$ that satisfies the constraints of (1)-(11). Take any $i = 1,\ldots,n_r$ and any $k = 1,\ldots,n_e$. We consider two cases depending on the value of y_i^k.

1. $y_i^k = 0$. From (3) we directly obtain $z_i^k \le 0$ and therefore $z_i^k = 0$.
2. $y_i^k = 1$. We consider two subcases:

- If for some $j \in \mathrm{R}_i$ we have $x_j^k = 0$ (a reagent is missing), then $z_i^k \le 0$ from (4) and therefore $z_i^k = 0$. Similarly, if for some $j \in \mathrm{I}_i$ we have $x_j^k = 1$ (an inhibitor is present), then from (5) $z_i^k \le 0$ and therefore $z_i^k = 0$.

- If for all $j \in \mathrm{R}_i$ we have $x_j^k = 1$ (all reagents present) and for all $j \in \mathrm{I}_i$ we have $x_j^k = 0$ (all inhibitors absent), then from (6) we obtain $z_i^k \geq 1$ and therefore $z_i^k = 1$.

Since the choice of i and k was arbitrary we have shown $Z \in \{0,1\}^{n_e \times n_r}$.

Relaxation of non-input x_j^k

For the case that no loops are present in the pathway, we will argue that we can also use $x_j^k \in [0,1]$ for all species but the input species. In typical pathways the majority of species are noninput species. The formal definition of input species is

Definition 1 (Input species) *Species j that are not products in any reaction, i.e.,* $\mathrm{T} \equiv \left\{ j \in \{1,\ldots,n_s\} : j \notin \cup_{i=1}^{n_r} \mathrm{P}_i \right\}$ are termed *input species*.

Theorem 2 *Suppose that the pathway proposed contains no loops. In (1)-(11) replacing $Z \in \{0,1\}^{n_e \times n_r}$ by $Z \in [0,1]^{n_e \times n_r}$ and $x_j^k \in \{0,1\}$ by $x_j^k \in [0,1]$ for all $j \notin \mathrm{T}$ (for all non-input species) is an exact reformulation, in the sense that any feasible point in the new program is also feasible in the original program.*

Note that input species cannot be relaxed, for otherwise $z_i \in \{0,1\}$ would not be ensured. The proof idea is that because the potential pathway form a directed graph, we can proceed from the "top" to the "bottom". In doing so we establish that both x_j^k and z_i^k are forced to be integer.

Proof. Take any $X \in [0,1]^{n_e \times n_s}$, $y \in \{0,1\}^{n_r}$ and $Z \in [0,1]^{n_e \times n_r}$ that satisfies the constraints of (1)-(11) and that also satisfies $x_j^k \in \{0,1\}$, for all $j \in \mathrm{T}$ (all input species are binary).

In the proof of Theorem 2 we have established that if for a given reaction i and experiment k, we have $x_j^k \in \{0,1\}$ for all $j \in \mathrm{R}_i \cup \mathrm{I}_i$ (all reagents and inhibitors are binary), then we also obtain $z_i^k \in \{0,1\}$.

Take $k \in \{1,\ldots,n_e\}$ (an arbitrary experiment) and $j \in \{1,\ldots,n_s\}$ (an arbitrary species). We will argue that if $z_i^k \in \{0,1\}$ for all $i \in \{1,\ldots,n_r\} : j \in \mathrm{P}_i$ (for all reactions for which the species is a product) then $y_j^k \in \{0,1\}$. There are essentially two cases:

1. If for some $i \in \{1,\ldots,n_r\} : j \in \mathrm{P}_i$ we have $z_i^k = 1$ then by (7) we obtain $x_j^k \geq 1$ and therefore $x_j^k = 1$.
2. If for all $i \in \{1,\ldots,n_r\} : j \in \mathrm{P}_i$ we have $z_i^k = 0$ then by (8) we obtain $x_j^k \leq 0$ and therefore $x_j^k = 0$.

It is clear that in the absence of loops the above two arguments propagate through the pathway. From an arbitrary species $j \in \{1, \ldots, n_s\}$ we can traverse the graph in reverse direction and reach the input species in a finite number of steps (a reverse path). Due to the absence of loops, each species depends only on the species which are “further up” in the pathway.
